# White - cGMP Interaction Promotes Fast Locomotor Recovery from Anoxia in Adult *Drosophila*

**DOI:** 10.1371/journal.pone.0168361

**Published:** 2017-01-06

**Authors:** Chengfeng Xiao, R. Meldrum Robertson

**Affiliations:** Department of Biology, Queen’s University, Kingston, Ontario, Canada; Biomedical Sciences Research Center Alexander Fleming, GREECE

## Abstract

Increasing evidence indicates that the *white* (*w*) gene in *Drosophila* possesses extra-retinal functions in addition to its classical role in eye pigmentation. We have previously shown that *w*^+^ promotes fast and consistent locomotor recovery from anoxia, but how *w*^+^ modulates locomotor recovery is largely unknown. Here we show that in the absence of *w*^+^, several *PDE* mutants, especially cyclic guanosine monophosphate (cGMP)-specific *PDE* mutants, display wildtype-like fast locomotor recovery from anoxia, and that during the night time, locomotor recovery was light-sensitive in white-eyed mutant w1118, and light-insensitive in *PDE* mutants under w1118 background. Data indicate the involvement of cGMP in the modulation of recovery timing and presumably, light-evoked cGMP fluctuation is associated with light sensitivity of locomotor recovery. This was further supported by the observations that *w*-RNAi-induced delay of locomotor recovery was completely eliminated by upregulation of cGMP through multiple approaches, including *PDE* mutation, simultaneous overexpression of an atypical soluble guanylyl cyclase Gyc88E, or sildenafil feeding. Lastly, prolonged sildenafil feeding promoted fast locomotor recovery from anoxia in w1118. Taken together, these data suggest that a White-cGMP interaction modulates the timing of locomotor recovery from anoxia.

## Introduction

The *Drosophila* *white* (*w*) gene encodes a hemi-unit of an ATP-binding cassette transporter (ABC transporter) which is proposed to transport a variety of small molecules including pigment precursors, 3-hydrokynurenine and bio-amines [1–6]. In addition to its classical role in eye pigmentation [7–9], increasing evidence indicates that the *w* gene has extra-retinal functions in the central nervous system (CNS) [4, 10–12]. We have recently shown that *w*^+^ is associated with fast and consistent locomotor recovery from anoxia in adult flies [13]. In fact, the high consistency of locomotion and electrophysiological performance has been a significant characteristic of wildtype Canton-S (CS) [14–19]. It is possible that the White protein possesses a housekeeping function in promoting appropriately timed release of second messengers or neurotransmitters and improving signaling efficacy during anoxic recovery. However, little is known about the specific substrates that might interact with White protein to promote fast and consistent locomotor recovery from anoxia.

In *Drosophila* larvae, cyclic guanosine monophosphate (cGMP) mediates escape responses from hypoxia [20]. cGMP can be transported by White protein into vesicles in the principle cells of Malpighian tubules [21]. These findings suggest a hypothesis that a White—cGMP interaction could occur in adult flies and mediate locomotor responses to anoxia. Behavioral modulations by cGMP are well-documented. Upregulation of cGMP increases ion conductance and depolarizes membrane potential in *Xenopus* oocytes, and promotes muscle contraction in *C. elegans* [22]. High levels of cGMP activate cGMP-dependent protein kinase and promote locomotion while feeding in larvae and adult flies [23, 24]. Increased cGMP promotes fast onset of anoxic coma via a cGMP-activated protein kinase [25]. In addition, genes for cGMP-specific phosphodiesterases (PDEs) have been identified in *Drosophila* [26, 27]. The PDEs rapidly hydrolyze cGMP upon light stimulation in the vertebrate phototransduction pathway [28–32]. Although cGMP is unlikely to be involved in *Drosophila* phototransduction [33–35], the existence of multiple cGMP-specific *PDE* genes as well as their expression in the CNS [26, 36] implies a functional conservation of PDEs in modulating cGMP levels in *Drosophila*. Therefore, there is a high probability that a White—cGMP interaction promotes fast and consistent locomotor recovery from anoxia.

Using multiple approaches, we examined the hypothesized White-cGMP interaction in the locomotor recovery from anoxia in adult flies. We show that several *PDE* mutants, especially cGMP-specific PDE mutants, display wildtype-like fast locomotor recovery, and that locomotor recovery of w1118 was light-sensitive in the night, which was abolished in *PDE* mutants. In addition, *Pde1c* mutation or simultaneous overexpression of an atypical guanylyl cyclase Gyc88E eliminated the delay of locomotor recovery induced by RNAi knockdown of *w*. Feeding with sildenafil, a potent inhibitor of cGMP-specific PDEs, suppressed the delay of locomotor recovery induced by RNAi knockdown of *w*, and promoted fast locomotor recovery in w1118.

## Materials and Methods

### Fly strains

Fly strains used for the experiments were CS (Bloomington stock center, #1), w1118 (L. Seroude laboratory). Other flies and their sources include: UAS-*w*-RNAi (#31088), UAS-Gyc88E (D.B. Morton laboratory), R50E11-Gal4 (#38750), and a collection of *PDE* mutants (listed in S1 Table).

Most *PDE* mutants were backcrossed into w1118 background for ten egenrations according to a previous protocol [37] except for *Pde9* mutants. The available *Pde9* mutants contain alleles on the X chromosome with a genetic background of *y*^1^*w*^67c23^ or *y*^1^w*. The *y*^1^*w*^67c23^ showed delayed locomotor recovery similar to that in w1118 (unpublished observations). Thus *Pde9* mutants were added into the tests without serial backcrossing. Each mutant contains a m*w*^+^-carrying transposon in the genome. Up to four copies of m*w*^+^ is insufficient to elicit fast and consistent locomotor recovery [13]. Therefore, *PDE* mutation largely accounts for the timing of locomotor recovery.

UAS-*w*-RNAi lines were backcrossed to *w*^+^;; TM3/TM6 to have target *w*^+^ involved. Firstly, male RNAi flies were crossed into *w*^+^;; TM3/TM6, and male progeny (*w*^+^/y;; UAS-*w*-RNAi/TM3) were backcrossed into *w*^+^;; TM3/TM6 for establishing *w*^+^;; UAS-*w*-RNAi stock.

All the flies were maintained on standard cornmeal medium at 21–23°C in 12h/12h of light/dark illumination (lights on at 7 am and off at 7 pm). Male flies were collected within two days after emergence, transferred into fresh food vials and subject to locomotor assay at least three days after collection. A period of 3-day free of anoxic exposure was guaranteed before locomotor assay. Tested flies were 3–9 days old.

### Locomotor assay

Locomotor assays were performed by following a previously described protocol [17]. Briefly, individual flies were loaded into small circular arenas (1.27 cm diameter and 0.3 cm depth). We have built a Plexiglas plate with 128 arenas (8 × 16) in an area of 31 × 16 cm^2^. The bottom side of plate was covered with thick filter paper, allowing air circulation. The fly plate was secured in a larger chamber (48.0 × 41.5 × 0.6 cm^3^). Room air or pure nitrogen gas was pumped in, homogenized and passed through 41.5 × 0.6 cm^2^ area at desired speed. We exposed flies to a 30 s anoxia (a flow of pure nitrogen at 10 L/min) after five min regular locomotion. Flies were then allowed 1 hr for recovery. Duration of anoxia exposure was pre-determined by preliminary tests. We observed that all the flies were immobilized within 10–15 s from the onset of anoxia. A flow of room air (2 L/min) was provided throughout the test except during the anoxic exposure.

These settings allow simultaneous examination of multiple strains or genotypes, each containing typically 8 or 16 flies. Locomotor activities were video-captured and the time to locomotor recovery was post-analyzed using custom-written fly tracking software [13, 17]. The locomotor assay was performed in a room with relative humidity of 60–70%.

### RT-PCR

RT-PCR was conducted to examine transcriptional changes in selected *PDE* mutants. Flies (20–30 of both sexes at 4–7 days old) were homogenized for RNA extraction using Ambion PureLink RNA mini kit (Cat. 12183020, Ambion). Total RNA (1 *μ*g) was reverse transcribed to cDNA by GoScript Reverse Transcription System (Cat. A5001, Promega). The target RNA variants and primers were: Pde1c-RC (5’-GAGCCAGCAGGAGTATCTGT-3’, 5’-CGAACGGAACTTGGGCTTCT-3’); Pde1c-RB,RD,RE,RG,RH,RJ,RK and RL (5’-GGATCATGAGACCGTTGCCT-3’, 5’-CGAACGGAACTTGGGCTTCT-3’); Pde6-RB, RC and RD (5’-GCCAGTGCCGAGGAGATAAT-3’, 5’-AACACAGAGCACCTTTCGCA-3’); Pde9-RB, RD, RE and RF (5’-CACTACAACGAGGTGCCCTT-3’, 5’-GTCCGCCACTTTGATCAGGA-3’). Transcript of ribosomal protein L32 (RpL32) was used as loading control with primers 5’-CACTACAACGAGGTGCCCTT-3’ and 5’-GTCCGCCACTTTGATCAGGA-3’. Taq DNA polymerase (MB101-0500, GeneDireX) was used for PCR.

### Light illumination

Most of the tests were performed under regular bright light illumination using a white light box (Logan portaview slide/transparency viewer) during the light time (between 10 am and 4 pm). The selected time window was three hours away from the morning and the evening peaks of locomotor activity [38]. A double layer of 600 nm filter film (Roscolux 26, Rosco Canada) was used to generate dim red illumination for some of the experiments. Part of the experiments was also conducted in the night between 10 pm and 4 am under either bright light or dim red. The specific light illumination for each test was described in the text.

### Sildenafil feeding

Sildenafil (PZ0003, Sigma-Aldrich) was prepared as a stock solution at 20 mM in dimethyl sulfoxide (DMSO). Sildenafil was added at 1:100 (v/v) to the food at around 60°C before hardening. The final concentrations of sildenafil were 0, 1, 10 or 100 *μ*M in the food. DMSO final concentration was controlled at 0.5% (v/v). In some of the tests, newly emerged male flies (0–2 days) were fed with the food containing sildenafil for four days (referred to as 4-day feeding). In other tests, feeding was started from embryos and lasted throughout the life cycle and four additional days after eclosion (life-cycle and 4-day feeding). Sildenafil-containing food vials were freshly prepared for 4-day feeding or life-cycle feeding. Locomotor recovery was examined in the light time under bright light illumination.

### Statistics

Because part of the data displayed non-Gaussian distribution, we presented data with scatter dot plot and box plot (box and Whiskers of Min to Max). Nonparametric Mann-Whitney or Kruskal-Wallis tests with Dunn’s multiple comparisons were performed to examine the difference of medians. *P* < 0.05 was considered significant differences between groups. The sample size for a typical locomotor assay was 8 or 16, or otherwise indicated.

## Results

### *PDE* mutants under w1118 background displayed fast locomotor recovery from anoxia

Although cGMP mediates behavioral responses to hypoxia in *Drosophila* larvae [20] and increased cGMP promotes fast onset of anoxic coma in adult flies [25], it is unclear whether cGMP modulates the speed of anoxic recovery. We tested this possibility by examining the time to locomotor recovery in *PDE* mutants under w1118 genetic background. Intracellular cGMP is hydrolyzed by the cGMP- or dual- specific (cGMP- and cAMP-specific) PDEs. A total of 24 *PDE* mutants (S1 Table), each of which carries a specific allele at the locus of either *Pde1c*, *Pde6*, *Pde8*, *Pde9* or *Pde11*, were examined.

Four out of a total of 24 *PDE* mutants displayed wildtype-like fast locomotor recovery from anoxia. Median time to recovery was 437.0 s (interquartile range (IQR) 344.0–496.0 s, n = 23) in *Pde1c*^KG05572^, 508.5 s (IQR 422.0–655.3 s, n = 16) in *Pde1c*^MI05025^, 581.5 s (IQR 506.5–674.3 s, n = 16) in *Pde6*^MB06146^, and 553.5 s (IQR486.0–787.5 s, n = 16) in *Pde9*^MI06972^. These four mutants showed similar time to recovery to CS (median 512.5 s, IQR 448.0–553.0 s, n = 16) with insignificant difference, but shorter time to recovery than w1118 (median 1015.0 s, IQR 755.3–1797.0 s, n = 16) (Kruskal-Wallis tests with Dunn’s multiple comparison) (S1 Table). The other tested *PDE* mutants displayed comparable or prolonged time to recovery compared with CS, and similar time to recovery to w1118. Mutant *Pde11*^e02198^ showed severely prolonged time to recovery compared to CS (*P* < 0.05, Kruskal-Wallis tests with Dunn’s multiple comparison) or w1118 (*P* < 0.05, Kruskal-Wallis tests with Dunn’s multiple comparison). Perhaps essential functions other than locomotor recovery were severely affected in *Pde11*^e02198^. It should be noted that mutant *Pde1c*^c04487^, which is associated with male sterility and mating behavioral defects [39], showed longer time to recovery than CS (*P* < 0.05, Kruskal-Wallis tests with Dunn’s multiple comparison). Whereas the product of *Pde1c* is dual-specific, both *Pde6* and *Pde9* encode cGMP-specific PDEs, indicating that cGMP-specific *PDE* mutations are sufficient for fast locomotor recovery. Taken together, four mutants (*Pde1c*^KG05572^, *Pde1c*^MI05025^, *Pde6*^MB06146^ and *Pde9*^MI06972^) displayed wildtype-like fast locomotor recovery from anoxia.

### Transcriptional downregulation in *PDE* mutants with wildtype-like locomotor recovery

**Fig 1:**
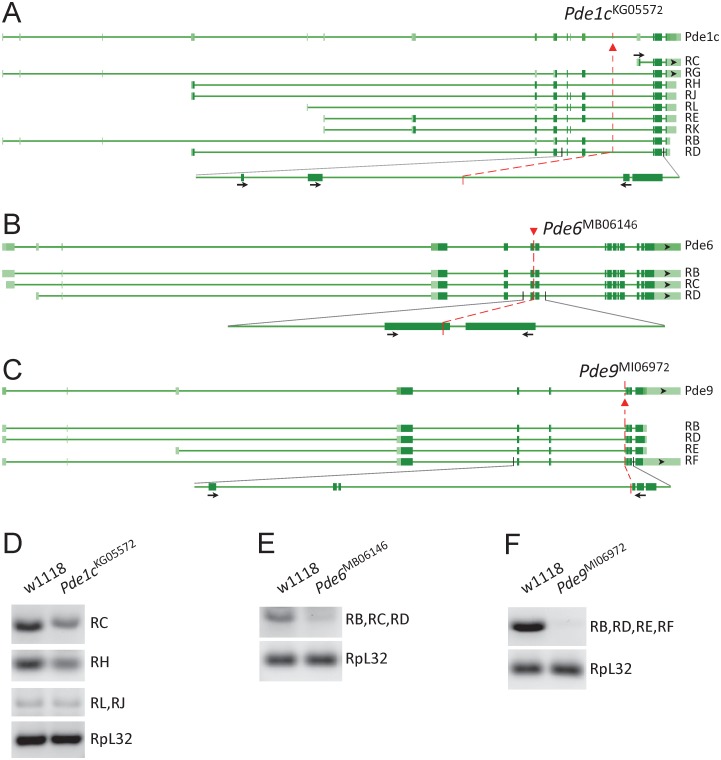
Transcriptional downregulation in *PDE* mutants. (A, B, C) Schematic gene structures and transcript variants for *Pde1c*, *Pde6* and *Pde9* generated from online NCBI’s sequence viewer. Mutants (*Pde1c*^KG05572^, *Pde6*^MB06146^, *Pde9*^MI06972^) and the sites for transposon insertion (red triangles) are illustrated. Part of the RNA regions are enlarged for the demonstration of locations of insertions (red dash lines) and PCR primers (black arrows). (D, E, F) RT-PCR results between control (w1118) and mutants. Transcriptional downregulation was observed in the mutants. RpL32: internal control.

We examined the changes of transcription in the selected *PDE* mutants that displayed wildtype-like locomotor recovery. Among nine variants of *Pde1c* messengers (Fig 1A), variant C (Pde1c-RC) and variant H (Pde1c-RH) were down-regulated in *Pde1c*^KG05572^ compared with w1118. Other individual variants including Pde1c-RL and RJ were likely unchanged (Fig 1D). Down-regulation of Pde1c-RC in *Pde1c*^KG05572^ was less than in *Pde1c*^c04487^ [39] due to different mutation sites. *Pde6* transcripts (Pde6-RB, RC and RD) were reduced in *Pde6*^MB06146^ (Fig 1B and 1E). *Pde9* transcripts (Pde9-RB, RD, RE and RF) were clearly down-regulated in *Pde9*^MI06972^ (Fig 1C and 1F). Thus, down-regulation of *PDE* transcription was observed in the mutants with the phenotype of wildtype-like locomotor recovery.

### Light sensitivity of locomotor recovery in w1118 in the night

**Fig 2:**
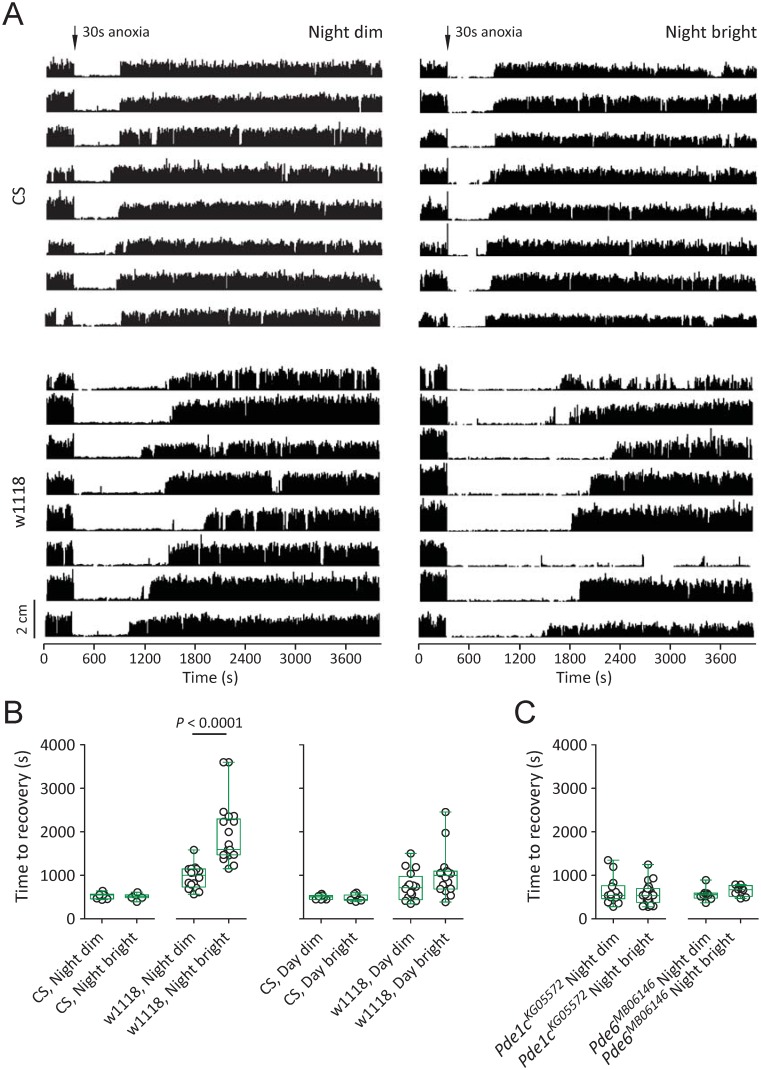
Light sensitivity of locomotor recovery from anoxia in w1118 in the night. (A) Locomotor analysis for CS and w1118 flies with anoxia. Each plot represents locomotor activity of a single fly. A 30 s anoxia (indicated as an arrow) was applied after 300 s locomotion under normoxia. Flies were then returned to normoxia and allowed to recover for 3600 s. (B) Light sensitivity of locomotor recovery in the night in w1118 flies. Time to recovery under two different illuminations (dim and bright) in the night or in the daytime were compared. Scatter dot plot (black circles) and box plot (median, box and Whiskers of min to max in green color) were shown. *P* value was obtained using Mann-Whitney test. (C) Elimination of light sensitivity of locomotor recovery in *PDE* mutants. Flies were tested under dim or bright in the night.

The cGMP level is subject to light-evoked changes and circadian fluctuations in vertebrates and invertebrates [31, 32, 40–43]. To examine the involvement of cGMP in locomotor recovery in *Drosophila*, we tested the possible light sensitivity of locomotor recovery. Two different illuminations, bright light and dim red (provided with a 600 nm long-pass filter), were applied. Dim red illumination mimics darkness because flies are poorly sensitive to red light [44]. In CS flies, median time to recovery under dim red was 549.0 s (IQR 461.0–566.5 s, n = 8), which was statistically the same as time to recovery under bright light (median 518.5 s, IQR 490.0–552.5 s, n = 8) (Fig 2A and 2B). In w1118, median time to recovery under dim red was 1005.0 s (IQR 733.3–1148.0 s, n = 16), a level significantly shorter than that under bright light (median 1595.0 s, IQR 1474.0–2297.0 s, n = 21) (*P* < 0.0001, Mann-Whitney test) (Fig 2A and 2B). Hence, locomotor recovery in w1118 but not CS displayed light sensitivity in the night.

During the daytime, time to recovery in CS under dim red (median 504.0 s, IQR 448.8–537.8 s, n = 8) was similar to that under bright light (median 447.0 s, IQR 427.3–551.5 s, n = 8) with no significant difference. Time to recovery in w1118 under dim red (median 717.0 s, IQR 441.5–977.5 s, n = 16) was also comparable with that under bright light (median 1001.0 s, IQR 690.5–1101.0 s, n = 16) with no statistical difference (Fig 2B). There was no light sensitivity of locomotor recovery in CS or w1118 during the daytime.

### Light sensitivity of locomotor recovery was abolished in *PDE* mutants

To further examine the involvement of cGMP in the modulation of locomotor recovery, light sensitivity of locomotor recovery in *Pde1c*^KG05572^ and *Pde6*^MB06146^ under w1118 background was examined. During the night time, time to recovery of *Pde1c*^KG05572^ flies under dim red (median 539.0 s, IQR 464.5–764.0 s, n = 14) was statistically the same as time to recovery under bright light (median 533.0 s, IQR 378.8–696.5 s, n = 22). Time to recovery of *Pde6*^MB06146^ under dim red (median 555.5 s, IQR 476.3–594.0 s, n = 8) was also the same as that under bright light (median 674.5 s, IQR 519.8–765.8 s, n = 8) (Fig 2C). Therefore, light sensitivity of locomotor recovery in the night was abolished in *Pde1c*^KG05572^ and *Pde6*^MB06146^ mutants.

### *Pde1c* mutation eliminated *w*-RNAi-induced delay of locomotor recovery

**Fig 3:**
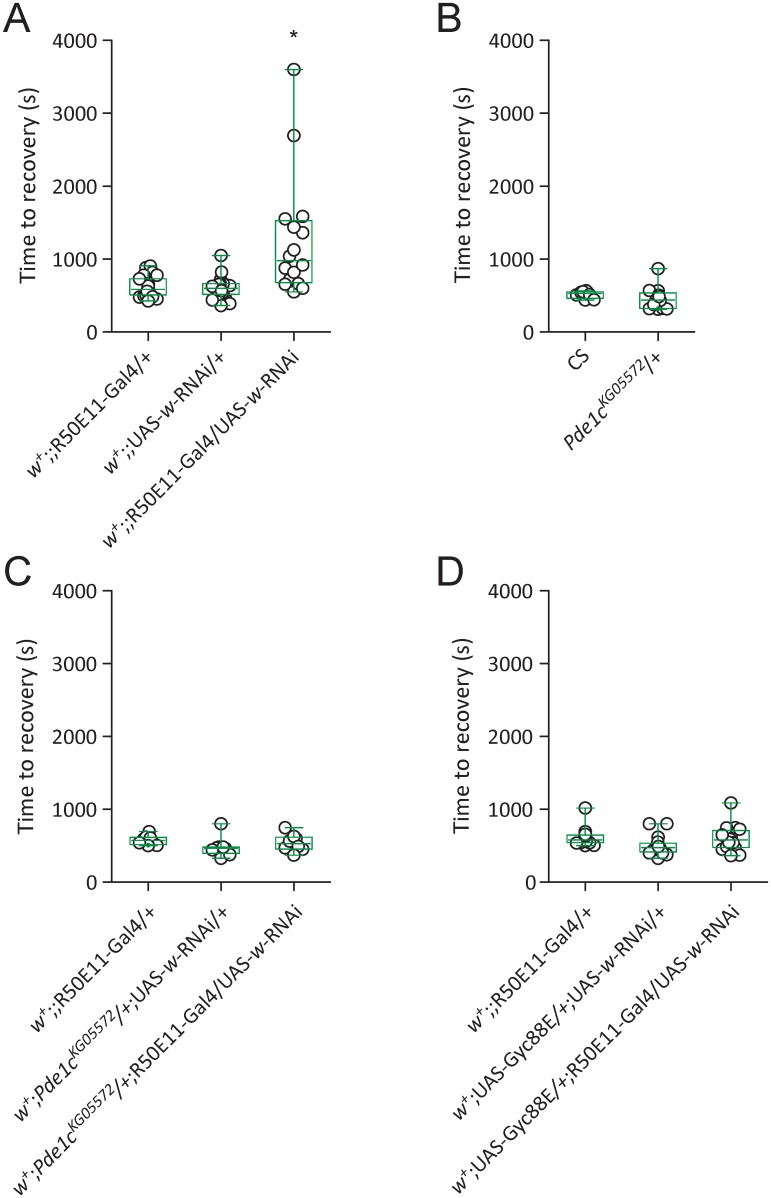
*Pde1c* mutation or Gyc88E overexpression suppresses *w*-RNAi-induced delay of locomotor recovery. (A) RNAi knockdown of *w* in serotonin subset delayed locomotor recovery from anoxia. * *P* < 0.05 by Kruskal-Wallis test with Dunn’s multiple comparison. R50E11-Gal4, driver specific for serotonin subset [13, 45]. (B) Time to recovery analysis in CS and w1118; *Pde1c*^KG05572^/+ flies. There was no significant difference of time to recovery between flies. (C) *Pde1c*^KG05572^ mutation suppressed *w*-RNAi-induced delay of locomotor recovery. (D) Simultaneous overexpression of Gyc88E suppressed *w*-RNAi-induced delay of locomotor recovery.

In addition to w1118, *w*^+^-carrying flies with RNAi knockdown of *w* in serotonin subsets show delayed locomotor recovery from anoxia [13]. We next examined whether *PDE* mutation could eliminate *w*-RNAi-induced delay of locomotor recovery. Time to recovery of *w*^+^;; R50E11-Gal4/UAS-*w*-RNAi (median 981.0 s, IQR 678.0–1528.0 s, n = 16) was longer than *w*^+^;; R50E11-Gal4/+ (median 588.0 s, IQR 508.0–731.0 s, n = 23) and *w*^+^;; UAS-*w*-RNAi/+ (median 602.0 s, IQR 518.0–663.5 s, n = 24) (*P* < 0.05, Kruskal-Wallis test with Dunn’s multiple comparison) (Fig 3A). Thus, RNAi knockdown of *w* in serotonin subset by R50E11-Gal4 delayed locomotor recovery from anoxia. This confirms results that we reported previously [13] but which are repeated here, from different experiments, to provide an appropriate comparison for the *PDE* mutant data.

Median time to recovery in flies carrying heterozygous *Pde1c* mutant allele (*Pde1c*^KG05572^/+) was 440.0 s (IQR 325.5–537.0 s, n = 13), which was similar to that in CS (median 530.0 s, IQR 461.8–553.0 s, n = 8) with no significant difference (Fig 3B). Hence, heterozygous *Pde1c*^KG05572^ allele was sufficient to promote fast locomotor recovery from anoxia.

Median time to recovery in *w*^+^; *Pde1c*^KG05572^/+; R50E11-Gal4/UAS-*w*-RNAi flies was 529.0 s (IQR 455.3–619.8 s, n = 8), which was statistically the same as time to recovery in *w*^+^;; R50E11-Gal4/+ flies (median 575.5 s, IQR 513.5–614.8 s, n = 8) or *w*^+^; *Pde1c*^KG05572^/+; UAS-*w*-RNAi/+ flies (median 465.0 s, IQR 395.5–481.8 s, n = 8) (Kruskal-Wallis test with Dunn’s multiple comparison) (Fig 3C). Therefore, *Pde1c* mutation eliminated *w*-RNAi-induced delay of locomotor recovery.

### Overexpression of atypical guanylyl cyclase Gyc88E eliminated *w*-RNAi-induced delay of locomotor recovery

To further address the potential role for cGMP upregulation in eliminating *w*-RNAi-induced delay of locomotor recovery, an atypical soluble guanylyl cyclase Gyc88E that normally catalyzes cGMP production under reduced oxygen levels [46], was simultaneously expressed in a *w*-RNAi targeted neuronal subset. Median time to recovery in flies (*w*^+^; UAS-Gyc88E/+; R50E11-Gal4/UAS-*w*-RNAi) was 578.5 s (IQR 476.3–708.8 s, n = 16), which was comparable with time to recovery in *w*^+^;; R50E11-Gal4/+ (median 579.0 s, IQR 542.5–646.3 s, n = 16) or *w*^+^; UAS-Gyc88E/+; UAS-*w*-RNAi/+ (median 474.5 s, 413.5–535.8 s, n = 16) (Kruskal-Wallis test with Dunn’s multiple comparison) (Fig 3D). Thus, simultaneous upregulation of cGMP by over-expressing Gyc88E in a serotonin subset was sufficient to eliminate delayed locomotor recovery induced by *w*-RNAi.

### Sildenafil eliminated delayed locomotor recovery induced by *w*-RNAi

**Fig 4:**
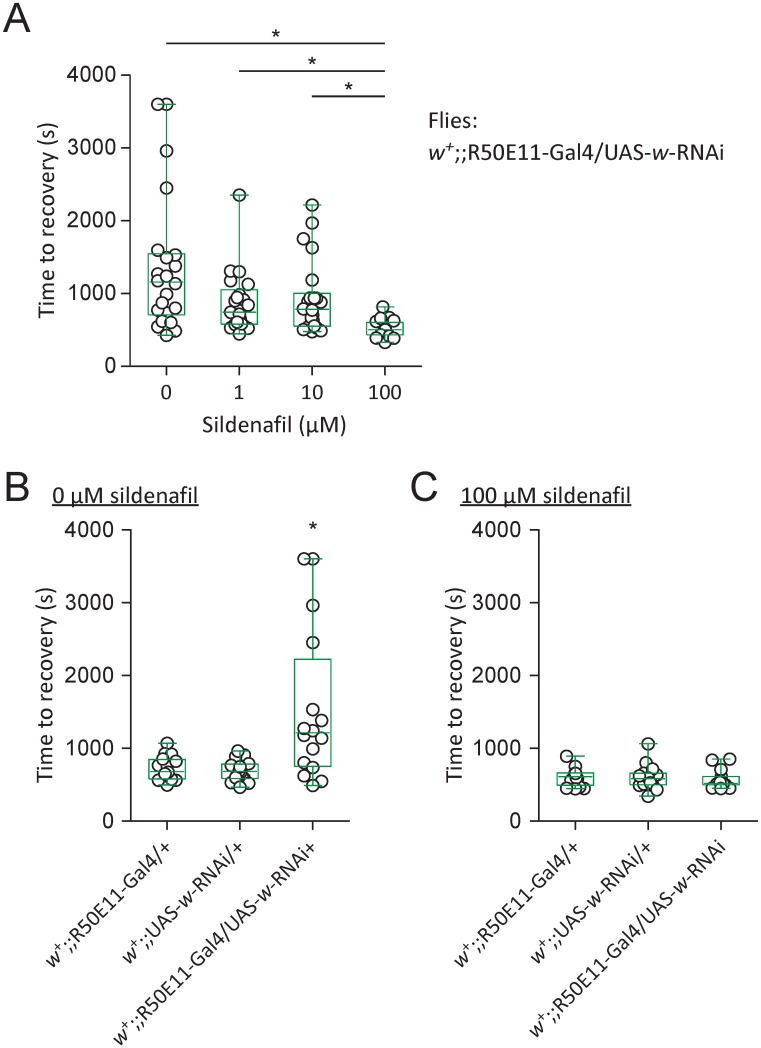
Sildenafil feeding suppresses *w*-RNAi-induced delay of locomotor recovery. (A) Time to recovery in flies (*w*^+^;; R50E11-Gal4/UAS-*w*-RNAi) fed with food containing 0, 1, 10 or 100 *μ*M sildenafil. * *P* < 0.05 by Kruskal-Wallis test with Dunn’s multiple comparisons. (B) Time to recovery in flies fed with vehicle control (containing 0 *μ*M sildenafil). Flies were fed for 4 days after eclosion. * *P* < 0.05 by Kruskal-Wallis test with Dunn’s multiple comparisons. (C) Time to recovery in flies fed with 100 *μ*M sildenafil. Feeding duration was the same as B.

Sildenafil, a specific inhibitor of vertebrate PDE5, blocks enzymatic activities of *Drosophila* PDE1, PDE6 and PDE11 [26]. There is a possibility that sildenafil would promote fast locomotor recovery through the inhibition of PDE activities with a consequence of cGMP upregulation. To test this idea, newly emerged male flies (*w*^+^;; R50E11-Gal4/UAS-*w*-RNAi) were fed with food containing sildenafil at 0, 1, 10 or 100 *μ*M for four days. Flies fed with 100 *μ*M sildenafil showed time to recovery (median 504.0 s, IQR 435.5–604.5 s, n = 21) shorter than that with 0 *μ*M (median 1158.0 s, IQR 707.3–1548.0 s, n = 22) (*P* < 0.05, Kruskal-Wallis test with Dunn’s multiple comparison), 1 *μ*M (median 744.0 s, IQR 581.5–1056.0 s, n = 21) (*P* < 0.05, Kruskal-Wallis test with Dunn’s multiple comparison) or 10 *μ*M (median 784.0 s, IQR 553.5–1004.0 s, n = 22) (*P* < 0.05, Kruskal-Wallis test with Dunn’s multiple comparison) (Fig 4A). Flies fed with 1 or 10 *μ*M sildenafil displayed the same time to recovery as vehicle control (0 *μ*M sildenafil). Thus, sildenafil feeding displayed an effect in a dosage-dependent manner to promote fast locomotor recovery.

We then examined whether sildenafil could eliminate delayed locomotor recovery induced by *w*-RNAi. Fed with vehicle control (0 *μ*M sildenafil) for four days, flies (*w*^+^;; R50E11-Gal4/UAS-*w*-RNAi) displayed time to recovery (median 1211.0 s, IQR 752.8–2221.0 s, n = 16) longer than *w*^+^;; R50E11-Gal4/+ flies (median 678.0 s, IQR 579.5–845.0 s, n = 16) (*P* < 0.05, Kruskal-Wallis test with Dunn’s multiple comparison) or *w*^+^;; UAS-*w*-RNAi/+ flies (median 682.5 s, IQR 584.3–781.3 s, n = 16) (*P* < 0.05, Kruskal-Wallis test with Dunn’s multiple comparison) (Fig 4B). Fed with 100 *μ*M sildenafil, however, *w*^+^;; R50E11-Gal4/UAS-*w*-RNAi flies displayed time to recovery (median 518.0 s, IQR 499.0–613.0 s, n = 15) similar to *w*^+^;; R50E11-Gal4/+ flies (median 609.0 s, IQR 491.0–656.8 s, n = 16) or *w*^+^;; UAS-*w*-RNAi/+ flies (median 581.0 s, IQR 499.0–658.0 s, n = 15) with no statistical difference (Fig 4C). Thus, feeding with 100 *μ*M sildenafil for four days eliminated delayed locomotor recovery induced by *w*-RNAi.

### Sildenafil promoted fast locomotor recovery in w1118

**Fig 5:**
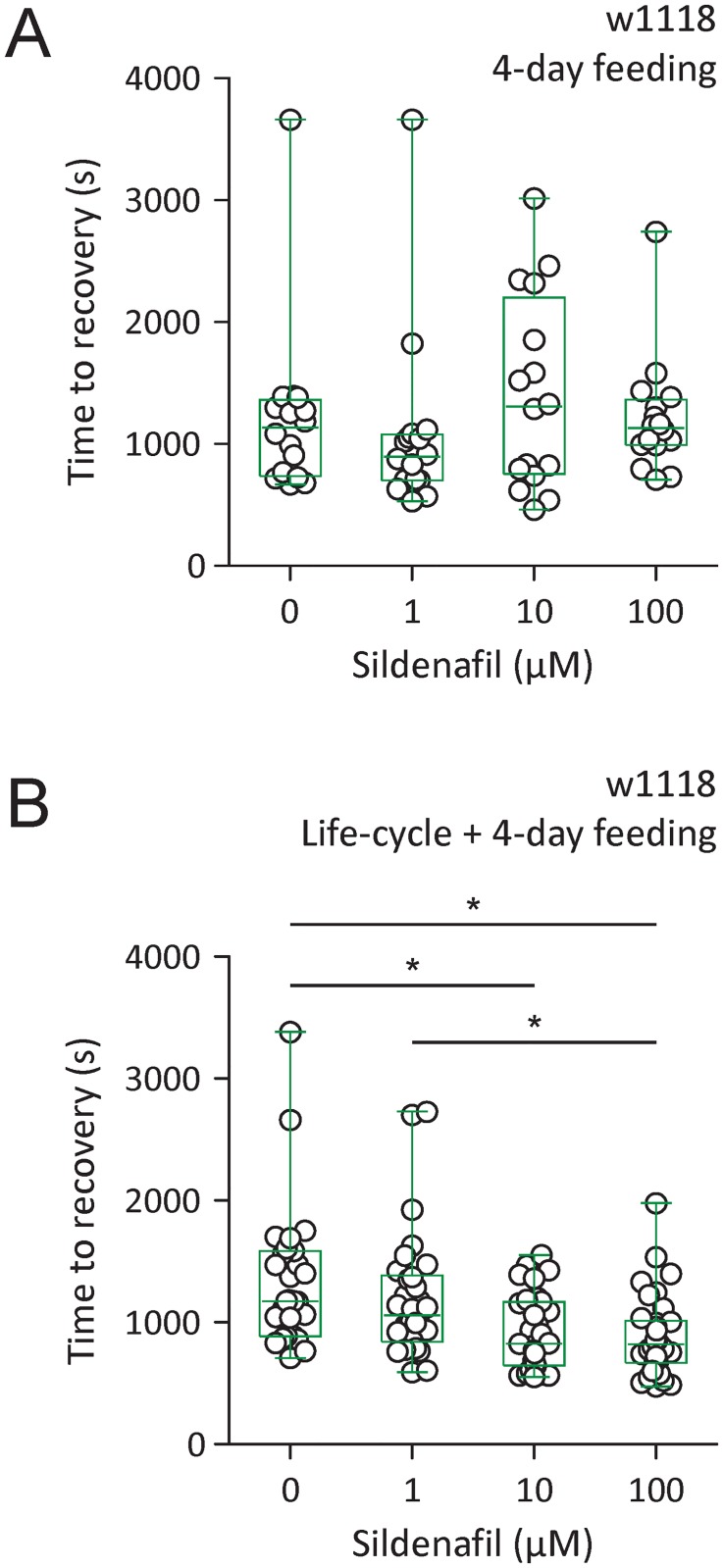
Prolonged sildenafil feeding promotes fast locomotor recovery in w1118. (A) Time to recovery analysis in w1118 fed with 0, 1, 10 or 100 *μ*M sildenafil for four days. The feeding started from eclosion. (B) Time to recovery analysis in w1118 fed with 0, 1, 10 or 100 *μ*M sildenafil for the entire life cycle and four additional days from eclosion. * *P* < 0.05 by Kruskal-Wallis test with Dunn’s multiple comparisons.

The effect of sildenafil in promoting fast locomotor recovery in w1118 was examined. Newly emerged w1118 flies fed with 100 *μ*M sildenafil for four days showed time to recovery (median 1133.0 s, IQR 991.0–1364.0 s, n = 16) similar to flies fed with 0 *μ*M sildenafil (median 1135.0 s, IQR 738.5–1362.0 s, n = 16), 1 *μ*M sildenafil (median 896.5 s, IQR 701.3–1079.0 s, n = 16), or 10 *μ*M sildenafil (median 1309.0 s, IQR 755.0–2203.0 s, n = 16) with no significant difference (Fig 5A). w1118 fed with 100 *μ*M sildenafil for a prolonged period (the entire life cycle and four additional days after eclosion) displayed time to recovery (median 821.0 s, IQR 667.8–1012.0 s, n = 34) comparable to flies fed with 10 *μ*M sildenafil (median 826.5 s, IQR 645.3–1167.0 s, n = 32), but shorter than flies fed with 0 *μ*M sildenafil (median 1174.0 s, IQR 886.0–15852.0 s, n = 27) (*P* < 0.05, Kruskal-Wallis test with Dunn’s multiple comparison) or 1 *μ*M sildenafil (median 1058.0 s, IQR 842.3–1385.0 s, n = 30) (*P* < 0.05, Kruskal-Wallis test with Dunn’s multiple comparison). w1118 flies fed with 10 *μ*M sildenafil for prolonged period also showed shorter time to recovery than flies fed with 0 *μ*M sildenafil (*P* < 0.05, Kruskal-Wallis test with Dunn’s multiple comparison) (Fig 5B). Therefore, prolonged feeding with 10 or 100 *μ*M sildenafil promoted fast locomotor recovery from anoxia in w1118.

## Discussion

Locomotor recovery from anoxia in adult *Drosophila* is complicated and little of the molecular basis is understood. We have previously shown that the *w* gene modulates the timing of locomotor recovery from anoxia [13]. Here we present data to support the hypothesis that a White—cGMP interaction promotes fast locomotor recovery. We find that several *PDE* mutants under w1118 background display wildtype-like fast locomotor recovery from anoxia, and that locomotor recovery in w1118 but not wildtype is light-sensitive in the night. upregulation of cGMP through multiple approaches, including *PDE* mutation, specific expression of Gyc88E, and sildenafil feeding, suppresses the *w*-RNAi induced delay of locomotor recovery. Finally, sildenafil feeding throughout the life cycle promotes fast locomotor recovery in w1118. Taken together, these findings suggest that a White-cGMP interaction promotes fast locomotor recovery from anoxia in adult *Drosophila*.

Fast and light-insensitive locomotor recovery in the night is observed in wildtype or *w*^1118^-carrying *PDE* mutants, indicating that cGMP upregulation without normal expression of White protein is sufficient to elicit wildtype-like performance of locomotor recovery. The product of mini-*white*^+^, carried in the transposon in each of selected *PDE* mutants, is insufficient for fast and consistent locomotor recovery [13]. These findings are further supported by the observation that cGMP upregulation through tissue-specific expression of Gyc88E in *w*-RNAi targeted cells completely eliminates the delay of locomotor recovery. Results imply that cGMP functions downstream of White protein in the modulation of recovery timing. The most plausible explanation is that the effect of cGMP upregulation is equivalent to the high efficacy of cGMP signaling in wildtype.

What could mediate the high efficacy of cGMP signaling in wildtype flies? Light-evoked cGMP hydrolysis by PDEs is one of the common characteristics in vertebrate phototransduction as well as in the CNS [28, 29, 31, 32, 40, 41]. The expression of PDEs in fly head [26, 36] and the minor importance of cGMP in *Drosophila* phototransduction [33, 34, 47] suggest a conserved cGMP signaling cascade in extra-retinal tissues. The *w* transcripts are detectable in the fly head [8, 10]. It is proposed that White protein possesses extra-retinal functions in additional to its classical role in eye pigmentation [4, 10–13]. The potential overlap between PDE/cGMP signaling and White expression in extra-retinal nervous system supports a White—cGMP interaction. In addition, White protein uptakes cGMP into vesicles in the Malpighian tubules in *Drosophila* [21]. In the fly head, White protein imports biogenic amines (histamine, dopamine and serotonin) as well as pigment precursors including 3-hydroxykynurenine into vesicles [1–3, 48, 49]. The biological significance for vesicular storage would be the avoidance of excessive enzymatic hydrolysis in the cytoplasm [21] and the promotion of synchronous release of vesicular contents. Together with our observations, these findings strongly support the notion that White imports cGMP and improves cGMP signaling in extra-retinal nervous system.

In vertebrate photoreceptors, cGMP infusion produces a rapid, large and persistent increase of light-sensitive current [30, 50, 51]. PDE-mediated circadian cGMP fluctuation is also common in mammalian circadian system [40, 41]. PDE activity is minimal during the day, and light pulse during the night modifies the cGMP levels in PDE-dependent manner [40]. In *Drosophila*, although the interaction between cGMP-dependent protein kinase (PKG) and circadian system is involved in the regulation of circadian feeding pattern [52], it is unclear whether cGMP itself or cGMP-specific PDEs are subjected to circadian modulations. Many of the clock genes in *Drosophila* display daily rhythms of transcription and post-translational phosphorylation [53–58]. Our observations that light sensitivity of locomotor recovery is present in the night but not in the daytime indicate the presence of circadian cGMP fluctuation in *Drosophila*.

The White—cGMP interaction in the modulation of locomotor recovery is further supported by the effects of sildenafil feeding. The delay of locomotor recovery is observed in w1118, or *w*^+^-carrying flies with *w*-RNAi targeted in a restricted cell population. Although sildenafil feeding promotes fast locomotor recovery in both flies, a relatively long feeding period is required for w1118 flies while a short period is sufficient for *w*^+^-carrying flies with targeted *w*-RNAi. Likely, partial loss or down-regulation of White protein poses tissue-specific and limited reduction of a White—cGMP interaction that could be relatively easier to rescue, whereas complete lack of White could require life-long treatment of sildenafil in order to elicit fast locomotor recovery. Sildenafil is a potent inhibitor of vertebrate PDE5 and *Drosophila* PDE6 [26], both of which are cGMP-specific. These data confirm the role for a White—cGMP interaction in the modulation of timing of locomotor recovery from anoxia.

## Supporting Information

S1 Table Time to locomotor recovery in *PDE* mutants after a 30 s anoxia. (PDF)
