# Supplementary material for: White - cGMP Interaction Promotes Fast Locomotor Recovery from Anoxia in Adult Drosophila
Source: PLoS One. 2017 Jan 6;12(1):e0168361. doi: 10.1371/journal.pone.0168361 (PMC5218474; doi:10.1371/journal.pone.0168361)
Supplement: S1 Table — (PDF) [file pone.0168361.s001.pdf]

White - cGMP interaction promotes fast locomotor recovery from anoxia in adult *Drosophila*

Chengfeng Xiao[1*], R Meldrum Robertson[1]

1 Department of Biology, Queen's University, Kingston, Ontario, Canada

* xiao.c@queensu.ca

Supporting Information

S1 Table. Time to locomotor recovery in *PDE* mutants after a 30 s anoxia.

Strains	n	Median (sec)	Interquartile range (sec)
CS	16	512.5	448.0 - 553.0
w1118	16	1015.0	755.3 - 1797.0
$Pde1c^{KG05572}$	23	437.0*	344.0 - 496.0
$Pde1c^{MI05025}$	16	508.5*	422.0 - 655.3
$Pde1c^{MB02790}$	14	559.0	484.8 - 855.0
$Pde1c^{MB03394}$	16	642.5	546.3 - 819.3
$Pde1c^{MB02052}$	15	647.0	551.0 - 855.0
$Pde1c^{MB00379}$	16	714.0	582.5 - 954.3
$Pde1c^{MB01415}$	16	833.5	676.3 - 1331.0
$Pde1c^{MB02304}$	10	1040.0	550.5 - 2270.0
$Pde1c^{MI04054}$	16	1063.0	799.8 - 1338.0
$Pde1c^{c04487}$	15	1158.0	813.0 - 1478.0
$Pde1c^{f02409}$	16	1384.0	700.3 - 3478.0
$Pde6^{MB06146}$	16	581.5*	506.5 - 674.3
$Pde6^{PL00055}$	15	1242.0	529.0 - 2020.0
$Pde6^{MI07106}$	16	2631.0	1063.0 - 3600.0
$Pde8^{EY10946}$	8	664.0	536.5 - 697.8
$Pde8^{08b}$	16	709.0	576.0 - 1207.0
$Pde8^{MI05352}$	16	843.5	561.0 - 1979.0
$Pde8^{EY10143}$	16	1253.0	660.8 - 1893.0
$Pde9^{MI06972}$	16	553.5*	486.0 - 787.5
$Pde9^{EY06288}$	24	731.0	598.3 - 906.5
$Pde11^{MI00079}$	16	1007.0	668.8 - 2946.0
$Pde11^{MI02899}$	16	1085.0	576.0 - 2552.0
$Pde11^{e03811}$	15	1460.0	672.0 - 2404.0
$Pde11^{e02198}$	16	3600.0	1953.0 - 3600.0

* Comparable to CS but shorter than w1118 (Kruskal-Wallis tests with Dunn's multiple comparison).
